# Vacuum-assisted closure increases ICAM-1, MIF, VEGF and collagen I expression in wound therapy

**DOI:** 10.3892/etm.2014.1567

**Published:** 2014-02-20

**Authors:** WEIYANG WANG, ZHENYU PAN, XIANG HU, ZONGHUAN LI, YONG ZHAO, AI-XI YU

**Affiliations:** Department of Micro-Orthopedics, Zhongnan Hospital of Wuhan University, Wuhan, Hubei 430071, P.R. China

**Keywords:** severe traumatic wound, vacuum-assisted closure, intercellular adhesion molecule-1, migration inhibitory factor, collagen I

## Abstract

Severe traumatic wounds are challenging to manage during surgery. The introduction of vacuum-assisted closure (VAC) is a breakthrough in wound management. The aim of the present study was to investigate the effect of VAC on cytokines in wounds during the management of severe traumatic wounds following initial debridement. VAC and conventional wound care (CWC) were independently applied to severe traumatic wounds on pigs. The expression levels of intercellular adhesion molecule-1 (ICAM-1), migration inhibitory factor (MIF), vascular endothelial growth factor (VEGF), basic fibroblast growth factor, collagen I and human fibroblast collagenase 1 were detected by quantitative polymerase chain reaction and western blotting. VAC significantly increased the expression of ICAM-1, MIF, VEGF and collagen I compared with that induced by CWC at the protein and mRNA levels. Therefore, the results of the present study indicate that VAC therapy is an effective method for treating severe traumatic wounds, as it increases the expression of cytokines in wounds. VAC significantly increases the expression of ICAM-1, MIF, VEGF and collagen I to manage severe traumatic wounds.

## Introduction

Severe traumatic wounds are challenging to manage during surgery and numerous methods of temporary wound closure have been reported. Presently, wounds are treated by conventional wound care (CWC) or by a vacuum-assisted closure (VAC) device. VAC may be applied in the majority of situations involving impaired wound healing. Firstly, VAC removes stagnant fluid and debris and then constantly optimizes blood supply and matrix deposition (1). Therefore, the partial oxygen pressure within the tissue increases and bacterial proliferation is reduced (2). Secondly, VAC results in increased local interleukin-8 and vascular endothelial growth factor (VEGF) concentrations, which may trigger the accumulation of neutrophils and angiogenesis (3). With the cyclical application of subatmospheric pressure, VAC alters the cytoskeleton of cells in the wound bed, triggering a cascade of intracellular signals that increase the rates of cell proliferation and division, and subsequent formation of granulation tissue (4).

With regard to the stimulation of vascularization and cell growth, VAC has been used in traumatic or non-traumatic soft tissue defects and postoperative wound infection (5,6). However, to the best of our knowledge, the effects of VAC on cytokine levels in severe traumatic wounds have not yet been investigated.

During wound healing, a robust inflammatory response starts immediately following any tissue damage. This is followed by a proliferation response that involves the migration and proliferation of keratinocytes, fibroblasts and endothelial cells. Subsequently, re-epithelialization and granulation tissue formation occurs and a scar finally forms (7). Intercellular adhesion molecule-1 (ICAM-1) is constitutively expressed at a low level by endothelial cells during these stages, but is rapidly upregulated during inflammation (8). Basic fibroblast growth factor (bFGF) is a key factor involved in wound healing (9,10). Macrophage migration inhibitory factor (MIF) is a cytokine with multiple functions within and beyond the immune system (11). In addition to the main function of inhibiting macrophage migration, MIF exhibits a broad range of immunostimulatory and proinflammatory activities (12).

Endothelial cells and fibroblasts play important roles in the proliferation response of wound healing. VEGF promotes neovascularization through the extension and growth of existing arterial and capillary networks. Fibroblasts are the primary source of collagen, on which wound strength significantly depends. Collagen I is the major collagen type in soft tissue and represents ~75% of collagens. Wiegand *et al* reported that protease and proinflammatory cytokine concentrations are elevated in chronic wounds compared with those in acute wounds and can be modulated by collagen I *in vitro* (13). Human fibroblast collagenase 1 (MMP-1) is the prototype for all interstitial collagenases (14). MMP-1 plays an important role in tissue morphogenesis and wound repair (14).

In the present study, severe traumatic wounds were created in a pig model and were treated with VAC or CWC. The expression levels of cytokines in the wound, including ICAM-1, MIF, VEGF, bFGF, collagen I and MMP-1, were determined for VAC- and CWC-treated wounds. These cytokines play essential roles in wound healing and are valuable for studying the mechanism of VAC in promoting the healing of severe traumatic wounds.

## Materials and methods

### Animal model

A total of eight healthy domestic pigs of either gender were purchased from the Central China Agricultural University (Wuhan, China). The pigs had an average body weight of 60 kg and were fasted overnight with water *ad libitum*. The experimental protocol was approved by the Ethics Committee for Animal Research at Wuhan University (Wuhan, China). All pigs received humane care in compliance with the Chinese Convention on Animal Care.

### Anesthesia

An intramuscular injection of 2 mg/kg xylazine (Bayer AG, Leverkusen, Germany) mixed with 20 mg/kg ketamine (Farmaceutici Gellini S.p.A., Aprilia, Italy) was used for premedication. Anesthesia was then induced with intravenous 4 mg/kg sodium thiopental (Pentothal; Abbott Scandinavia AB, Solna, Sweden) and 2 μg/kg fentanyl (Leptanal; Lilly France S.A.S., Fegersheim, France). The pigs were administered a continuous infusion of 3.5 μg/kg/h fentanyl in Ringer’s acetate combined with intermittent bolus doses of 2.5 mg/kg sodium thiopental. Cuffed endotracheal tubes were then orally inserted. Mechanical ventilation was established with a Siemens-Elema ventilator (Siemens-Elema AB, Solna, Sweden) in a volume-controlled mode (65% N_2_O, 35% O_2_). The ventilatory settings were identical for all pigs (respiratory rate, 15 breaths per min; minute ventilation, 12 liters per min). A positive end-expiratory pressure of ~5 cm H_2_O was applied. A Foley catheter was intubated into the bladder by suprapubic cystotomy.

### Surgical procedure and tissue collection

A circular wound measuring 3 cm in diameter and 2 cm in depth, passing through the subcutaneous and muscle tissues, was created on the back of the pigs. A saline-soaked AMD gauze [Kendall Healthcare (Covidien), Mansfield, MA, USA] was used as wound filler. The gauze volume was ~1.5 times larger than the wound volume to allow for volume reduction during negative-pressure application. A drainage tube was inserted into the gauze and connected to a vacuum source (Prospera PRO-III; Prospera Technologies, LLC., Fort Worth, TX, USA). The wound was then sealed with a transparent adhesive drape (KCI Medical ApS, Ballerup, Denmark) that overlapped the wound margins by 10 cm. Following the completion of the experiments, the pigs were sacrificed with a lethal dose of intravenous 60 mM potassium chloride (Farmaceutici Gellini S.p.A.). The VAC group (Wuhan VSD Medical Science and Technology, Co., Ltd., Wuhan, China) was treated with a continuous negative pressure of 125 mmHg. The foam and dressings were changed under clean conditions every 5–7 days depending on the wound volume (a greater wound volume resulted in fewer days that the VAC held). Suction was turned off for 30–60 min prior to changing the VAC dressing. The contralateral wounds, named as the CWC group, were treated with a moist gauze. Dressings were changed every other day and visible debris was cleaned with a cotton-based tissue. A transparent adhesive drape was then used to overlap the wound margins by 5 cm. Tissues from the VAC and CWC groups were collected by removing viable tissue from the center of the wound. Following tissue collection, the pigs were sacrificed with a lethal dose of potassium chloride that was injected into the heart.

### RNA extraction and cDNA synthesis

For quantitative polymerase chain reaction (qPCR), ~100 mg specimen was washed with phosphate-buffered saline (PBS) prior to RNA extraction. Total RNA was extracted with TRIzol reagent (Invitrogen Life Technologies, Carlsbad, CA, USA). RNA concentration was determined by absorbance measurements at 260 nm, while the purity was determined by the ratio of the absorbance at 260 and 280 nm (260/280 ratio) with a BioPhotometer (Eppendorf, Hamburg, Germany). Reverse transcription with ~1 μg RNA was performed with random primers using a ReverTra Ace kit (Toyobo Co., Ltd., Osaka, Japan). The mRNA expression levels of the target genes (ICAM-1, MIF, VEGF, bFGF, collagen I, and MMP-1) and internal control gene, β-actin, were quantified with a qPCR detection system (SLAN Real-Time PCR system; Shanghai Hongshi Medical Technology, Co., Ltd., Shanghai, China) with SYBR Green I (Toyobo Co., Ltd.). PCR was performed according to standard procedures following optimization and was within the exponential range of amplification (15). The primer sequences were analyzed by the nucleotide basic local alignment search tool for specific gene amplification (16). The process was conducted with omission of a cDNA template as a negative control. Triplicate measurements were performed for all genes per subject and the mean data were used. For the relative quantification of the gene expression level, standard curves were constructed by considering at least three points of a 10-fold dilution series of cDNA in water. Relative gene expression data are expressed as the n-fold change in the transcription of the target genes normalized against the endogenous control in the same sample.

### Protein extraction and western blotting

For protein extraction, ~100 mg frozen granulation tissue was ground in liquid nitrogen, washed twice with ice-cold PBS, lysed in 1 ml radioimmunoprecipitation assay (RIPA) lysis buffer (Santa Cruz Biotechnology, Inc., Santa Cruz, CA, USA) and then sonicated on ice.

The lysates were collected and centrifuged at 12,000 × g for 10 min at 4°C. Proteins in the supernatants were collected and stored at −80°C until the concentration was analyzed with a bicinchoninic acid (BCA) protein assay kit (Shanghai Sangon Biological Engineering Technology & Services Co., Ltd., Shanghai, China). Following heating at 99°C for 5 min in a loading buffer, equal volumes of the tissue lysates (40 μg protein) were loaded for analysis by sodium dodecyl sulfate polyacrylamide gel electrophoresis and subsequently electrotransferred from the gels onto polyvinylidene difluoride membranes (Millipore Corporation, Billerica, MA, USA). The transferred membranes were blocked with 5% skimmed milk in Tris-buffered saline with 0.05% Tween (TBST) and washed six times in TBST. Target proteins (ICAM-1, MIF, VEGF, bFGF, collagen I, and MMP-1) were detected with anti-target protein and anti-β-actin monoclonal antibodies (mAbs; Santa Cruz Biotechnology, Inc.), which were diluted according to the manufacturer’s instructions and incubated overnight at 4°C. This was followed by incubation with peroxidase-conjugated goat anti-rabbit immunoglobulin (1:2,000; Santa Cruz Biotechnology, Inc.) in TBST for 1 h. Signals were developed using an enhanced chemiluminescent reagent (Pierce Biotechnology, Inc., Rockford, IL, USA) and β-actin was used as an internal loading control. Band intensity was analyzed using Quantity One software (Bio-Rad Laboratories, Inc., Hercules, CA, USA). Relative expression was calculated as the intensity ratio of the target protein to that of β-actin.

### Immunohistochemistry

Sections were cut from formalin-fixed, paraffin-embedded granulation tissue biopsies and hydrated through graded alcohols. For antigen unmasking, the sections were treated in trypsin solution for 10 min at 37°C. The sections were then washed with deionized water and incubated with 3% H_2_O_2_ for 5 min. The sections were incubated in the primary polyclonal antibody, anti-CD31, at 1:200 (Santa Cruz Biotechnology, Inc.) or anti-VEGF at 1:200 (Santa Cruz Biotechnology, Inc.) for 1 h at room temperature. Next the sections were incubated with secondary antibodies and peroxidase-conjugated streptavidin-biotin complex (Santa Cruz Biotechnology, Inc.) at 37°C for 30 min. Immunoreactivity was visualized with diaminobenzidine (Zymed Laboratories, Inc., South San Francisco, CA, USA). Negative controls were prepared by omitting the primary antibody.

### Vessel density following VAC and CWC

VAC and CWC wound biopsies (20 cases each) were randomly selected and then investigated by an experienced pathologist who was blinded to the type of wound dressing. The vessels were highlighted by CD31 that was counted per 1 mm^2^. The area with the highest vessel density was selected when the vessel density in the biopsy was heterogeneous. Experiments were performed twice.

### Statistical analysis

All statistical analyses were performed using SPSS version 13.0 software for Windows (SPSS, Inc., Chicago, IL, USA). To analyze the target genes and proteins in the VAC or CWC treatment groups, one-way repeated-measure analysis of variance was conducted. Statistical analysis was performed using the Mann-Whitney U test or the Student’s t-test, following Levine’s test for equality of variances, to compare wound cytokine expression in granulation tissue samples between the VAC and CWC treatment groups. The Mann-Whitney U test was used for non-normal continuous variables. Vessel density and VEGF expression in VAC- and CWC-treated wounds were analyzed with the Student’s t-test and the Mann-Whitney U test, respectively. P<0.05 was considered to indicate a statistically significant difference.

## Results

### ICAM-1, MIF, VEGF, bFGF and collagen I mRNA expression levels are higher in the VAC group than in the CWC group

**Figure 1:**
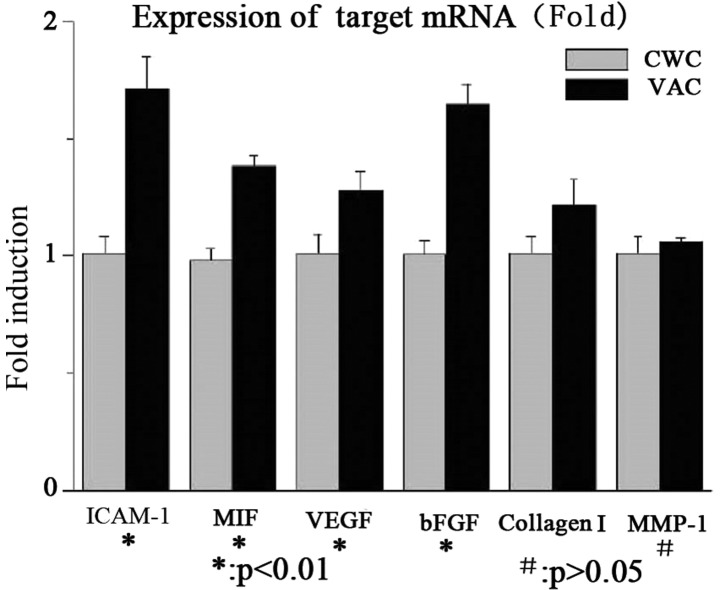
mRNA expression levels of cytokines in wounds treated with VAC or CWC (fold change). The mRNA expression levels of ICAM-1, MIF, VEGF, bFGF and collagen I were significantly higher in the VAC group than in the CWC group (P<0.01). No significant difference was identified in the MMP-1 mRNA expression levels between the two groups (P>0.05). VAC, vacuum-assisted closure; CWC, conventional wound closure; ICAM-1, intercellular adhesion molecule-1; MIF, migration inhibitory factor; VEGF, vascular endothelial growth factor; bFGF, basic fibroblast growth factor; MMP-1, human fibroblast collagenase 1.

The mRNA expression levels of ICAM-1, MIF, VEGF, bFGF and collagen I were significantly higher in the VAC group than in the CWC group. No significant difference was observed in the MMP-1 mRNA expression levels between the two groups. The results are summarized in Fig. 1.

### Protein expression levels of ICAM-1, MIF, VEGF and collagen I are higher in the VAC group than in the CWC group

**Figure 2:**
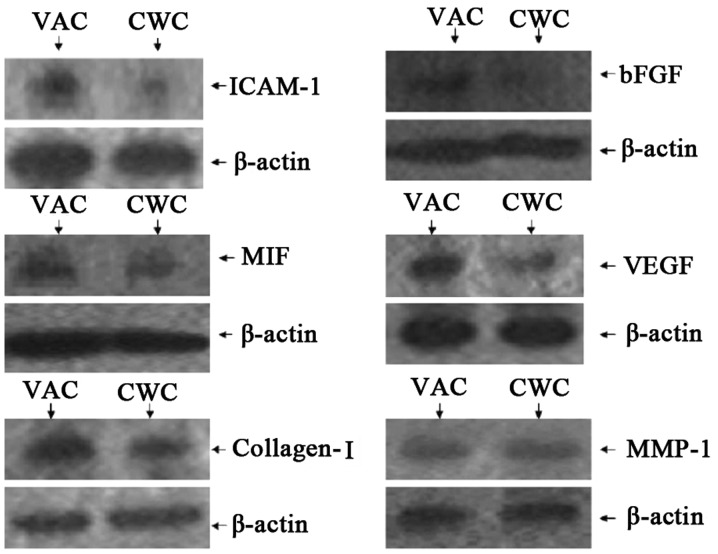
Protein expression levels of cytokines in wounds treated with VAC and CWC. ICAM-1, MIF, VEGF and collagen I were expressed at a higher level in the VAC group than in the CWC group at the protein level. However, bFGF was expressed at a very low level compared with β-actin in the VAC and CWC groups (P<0.01). No significant difference was observed in MMP-1 protein expression levels between the two groups (P>0.05). VAC, vacuum-assisted closure; CWC, conventional wound closure; ICAM-1, intercellular adhesion molecule-1; MIF, migration inhibitory factor; VEGF, vascular endothelial growth factor; bFGF, basic fibroblast growth factor; MMP-1, human fibroblast collagenase 1.

**Figure 3:**
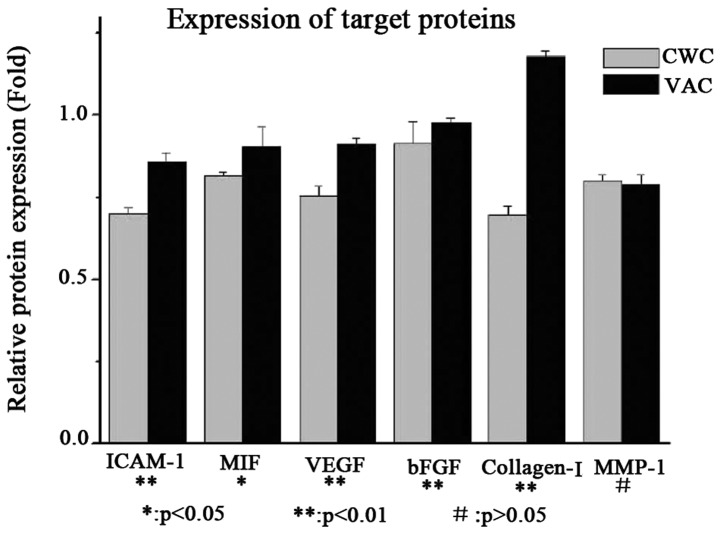
Protein expression levels of cytokines in wounds treated with VAC and CWC (analysis). ICAM-1, MIF, VEGF and collagen I were expressed at a higher level in the VAC group compared with the CWC group at the protein level. However, bFGF was expressed at a very low level compared with β-actin in the VAC and CWC groups (P<0.01). No significant difference was observed in MMP-1 protein expression levels between the two groups (P>0.05). VAC, vacuum-assisted closure; CWC, conventional wound closure; ICAM-1, intercellular adhesion molecule-1; MIF, migration inhibitory factor; VEGF, vascular endothelial growth factor; bFGF, basic fibroblast growth factor; MMP-1, human fibroblast collagenase 1.

The protein expression levels of ICAM-1, MIF, VEGF and collagen I were observed to be higher in the VAC group than in the CWC group. However, bFGF was expressed at a very low level compared with β-actin in the VAC and CWC groups. No significant difference in the levels of MMP-1 protein expression was observed between the two groups (Figs. 2 and 3).

### Vessel density following VAC and CWC treatment

**Figure 4:**
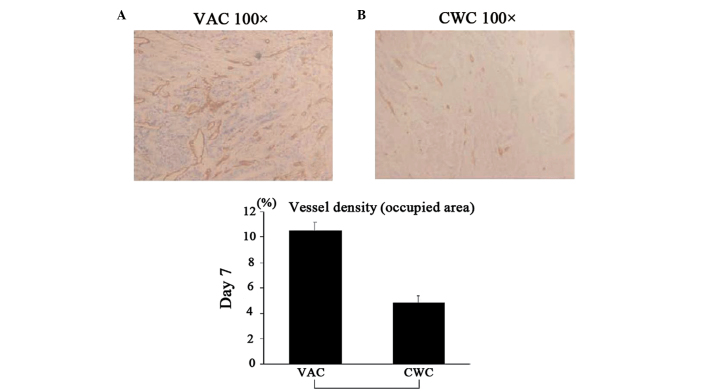
Vessel density in wounds treated with (A) VAC and (B) CWC. Vessel density was determined as the percentage of CD31-positively stained areas using PhotoShop (magnification, ×100). Vascular density in the wound bed of the VAC group was significantly higher compared with that in the CWC group (P<0.01). VAC, vacuum-assisted closure; CWC, conventional wound closure.

To assess the extent of angiogenesis, immunohistochemical staining with anti-CD31 mAbs was performed. The vascular density in the wound bed of the VAC group was significantly higher compared with that in the CWC group (Fig. 4).

### Expression of VEGF in wound biopsies following VAC and CWC

**Figure 5:**
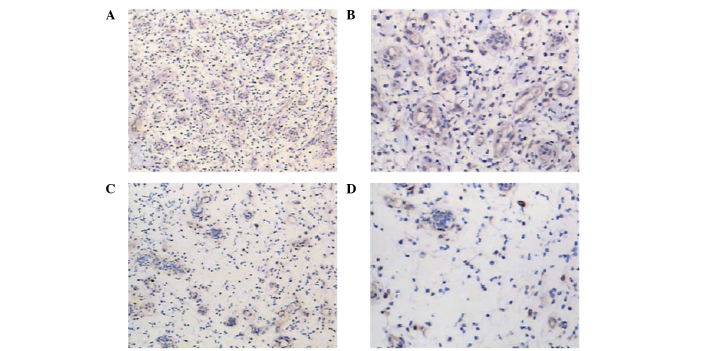
Immunohistochemistry of VEGF in wounds treated with VAC or CWC. VEGF expression in wounds treated by (A) VAC (magnification, ×100), (B) VAC (magnification, ×200), (C) CWC (magnification, ×100) and (D) CWC (magnification, ×200). VAC, vacuum-assisted closure; CWC, conventional wound closure; VEGF, vascular endothelial growth factor.

Immunohistochemical analysis revealed that the wounds in the VAC group showed visibly higher expression levels of VEGF (Fig. 5A and B) when compared with the levels in the CWC group (Fig. 5C and D). This was statistically verified by semi-quantitative analysis (P<0.005).

Macroscopic images of the wounds were captured by photography under the same conditions and the sizes of the wounds were digitally measured. Photographs of the wounds were taken with a digital camera (Canon, Beijing, China) and we measured the length and width of the wounds with a digital vernier caliper (NSCING Co., Ltd., Nanjing, China) every time the dressing were changed. Similarly to the study by Morykwas *et al* (17), compared with the CWC-treated wounds, the VAC-treated wounds in the present study were significantly drier and were larger on day 7, but were significantly smaller and closed faster on day 14 (data not shown). Re-epithelialization started immediately following injury from the wound edge to cover the denuded site.

## Discussion

Wounds are a major public health issue worldwide. One novel therapeutic approach to treating wounds is VAC therapy, which has become the most common treatment for severe traumatic wounds in Zhongnan Hospital.

Numerous theories have been put forward to explain the marked improvement in clinical outcomes achieved by VAC. Firstly, the application of suction in the VAC device removes interstitial fluid and cellular debris, reduces local edema and decreases the probability of wound infection (18). Secondly, the resulting hypobaric pressure and increase in blood flow to the wound bed accelerates vascularization and granulation-tissue formation (19,20). However, whether the cytokines present in the wound are involved in the VAC-assisted wound therapy remains unknown.

ICAM-1 is constitutively expressed at a low level by endothelial cells, but is rapidly upregulated during inflammation in wound healing (8,21). Nagaoka *et al* (22) reported that a lack of ICAM-1 delays wound healing, which is associated with the decreased infiltration of neutrophils and macrophages. In the present study, ICAM-1 was expressed in greater amounts at the mRNA and protein levels on day 7 of wound healing following VAC, when compared with those following CWC. VAC treatment increased ICAM-1 expression in 7 days, which indicates that VAC promotes the inflammation of wound healing, based on the higher ICAM-1 expression levels. bFGF has been shown to induce leukocytes to infiltrate wounds in significant numbers, although the mechanism remains unclear (10). In addition, bFGF promotes fibroblast proliferation, neovascularization and keratinocyte migration, which accelerates wound healing (23). In the present study, the mRNA expression level of bFGF was higher following VAC treatment compared with that following CWC. However, bFGF protein was expressed at low levels in the two groups and exhibited a significant difference between the groups.

MIF is a potent cytokine with multiple functions within and beyond the immune system (11). In addition to the main function of inhibiting macrophage migration, MIF has a broad range of immunostimulatory and proinflammatory activities (12). MIF also exhibits proangiogenic activity through the direct induction of VEGF, which is a representative angiogenic factor in wound healing (24,25). The results of the present study revealed the upregulation of MIF in VAC-treated wounds, when compared with the MIF level in CWC-treated wounds. In addition, MIF positively correlated with VEGF expression at the mRNA and protein levels. In accordance with a previous study, the basal expression of VEGF increased on day 7 of wound healing (26). Accordingly, the vessel density that was signified by immunochemistry with CD31 confirmed the hypothesis regarding the effect of VAC on neovascularization.

Collagens play an important role in wound repair and their deposition primarily determines the quantity of fibronectin and the quality of wound (27). Collagen type I represents 75% of collagens (13,28). MMP-1 is considered to be the prototype of all the interstitial collagenases and has been shown to play an important role in tissue morphogenesis and the formation of hypertrophic scars in wound repair (29,30). MMP-1 cleaves collagen I, II and III at specific sites on their α chains (31). It has been reported that bFGF increases collagen degradation and reduces scar formation by upregulating MMP-1 expression (4). Considering that MMP-1 degrades collagens and bFGF reduces collagen deposition and consequently suppresses scar formation, bFGF and MMP-1 may play an anti-scarring role in wound repair (1–4,18–20,30). In the present study, the expression level of collagen I was higher in the VAC group than in the CWC group, which is consistent with the clinical observations. MMP-1 expression also exhibited no significant difference between the two groups on day 7 of wound healing.

The results of the present study indicate that the time of secondary surgical procedures was significantly shorter in the VAC group than in the CWC group. In addition, the duration of treatment until complete closure of the wound was achieved was significantly shorter. Whelan *et al* (4) demonstrated that VAC removes excessive fluids containing bacteria equally throughout the wound, which enhances neovascularization and accelerates the formation of granulation tissue. This finding confirmed the observation of the present study that improved wound healing care of severe traumatic wounds was achieved following VAC therapy compared with CWC application.

In addition, it was identified that changes in VAC dressings were more costly than changes in CWC and that VAC required more intensive care compared with CWC. However, VAC required less overall attention than CWC due to the significantly lower number of repeat debridements and shorter duration of in-patient care.

In summary, the present study indicated that VAC significantly increased the expression of ICAM-1, MIF, VEGF and collagen I compared with the levels induced by CWC treatment. VAC was found to accelerate inflammation and neovascularization and to promote collagen deposition. The duration of treatment until complete wound closure was achieved was significantly shorter in the VAC group. Furthermore, VAC appears to be a safe procedure since no major complications occurred. Therefore, VAC provides critical assistance for the treatment of severe traumatic wounds.
